# Research education in Europe: an opinion paper by the European Society of Radiology

**DOI:** 10.1007/s13244-015-0397-x

**Published:** 2015-03-13

**Authors:** European Society of Radiology (ESR)

**Affiliations:** Neutorgasse 9/2, 1010 Vienna, Austria

## Abstract

Research is a major positive driver for radiology. Therefore, research education needs to be a major topic for the radiology leadership, including the research committee of the ESR. Professional (radiological and non-radiological) and scientific publications as well as Research Committee questionnaires provide the basis for this opinion paper. Although radiology is well-positioned to deal with current and future challenges, there are still some gaps, such as the presence of radiology in basic research, radiology-specific research versus research services for other disciplines, need of adaptation to new research topics, general attitude towards research, issues of career planning, lack of incentives for researchers, gender issues with loss of women from the researcher pipeline, limited financing of research education and variability between countries and institutions. There is no easy answer to such challenges. However, all stakeholders, from the ESR to subspecialty societies, university departments, general radiology departments and the individual radiologist must recognise and promote research within their competencies. Many means and structures are already available but need to be used more extensively and systematically. Additional means need to be developed, scientific and professional trends must be actively followed, and minimal standards in research education should be maintained throughout Europe.

*Main Messages*

*• Radiology research includes a broad spectrum, from basic to health services research.*

*• Research education needs to be widely available and systematically promoted.*

*• Existing means such as the European Institute for Biomedical Imaging Research (EIBIR) need to be advanced.*

*• New developments in research topics and professional life must be continuously monitored and evaluated.*

## Introduction

Radiology is a research-driven discipline. It needs to remain so and will expand into new research areas. Radiology covers basic scientific research, technical developments, applied clinical research and health services research in order to contribute to patient wellbeing, referring physicians, healthcare organisations and society. Evidence-based medicine has not yet played a very important role in radiology, but is here to stay [1] and will be applied by policy makers in most countries. Currently, the evidence for radiology practice is largely unsupported by high-level evidence and pays little heed to patient outcome.

To limit ourselves to clinical applications will not be sufficient. Disciplines contributing to basic research will have the best opportunity to apply and evaluate new knowledge early. Such knowledge promotes teaching. Those who teach will be recognised as the intellectual owners of procedures and diagnostic tools and are likely to be asked to advise on protocols, guidelines and in remuneration schemes. All this could be taken over by professionals from other disciplines, many of whom already have well-funded research activities, strategic plans and substantial expertise.

Research is a positive driver for radiology for other reasons, too, as it attracts the best staff, is the basis for third party financing and opens new business opportunities. This does not mean that the hard working, well-trained, clinical radiologist should be sidelined. He or she fulfils a very important role for patients, clinical departments, hospitals and the healthcare system. Their work may support at least part of research infrastructure and equipment and provides clinical data essential for many types of research. However, to recruit, motivate and provide consistent career planning for researchers is especially demanding and requires special attention from radiology leaders.

This White Paper is not a general statement regarding research but rather concentrates on research education. We have more than enough reason to plan research education, which is an important prerequisite when recruiting and maintaining research-oriented staff, allowing them to perform high-quality research and in order to be credible partners for clinical colleagues and other stakeholders in medical research.

After the initial training phase, there is a brain drain into non-academic settings where payment is often better and work is more compatible with family life or dual careers. Gender issues are commonly perceived as an additional burden to department leaderships but also represent a strength considering the input of diverse opinions and attitudes.

This paper analyses gaps in current research education. The analysis is based on two questionnaires sent to national and specialty societies throughout Europe, one of which has been published in *Insights into Imaging* [2]. Current educational activities include several means to promote research in radiology, many of which have already been applied successfully in single institutions, individual countries or even throughout Europe, but probably not in a systematic fashion. For future solutions, many of the existing programmes may be further developed, expanded or concentrated, broadened or focused, be combined with other activities, be better promoted, or become better accessible, for instance by using teleconferencing. However, many institutions and individuals will contribute to research and research education, including the individual European states and their institutions and societies, European regulations, societies, their committees, individual universities, university hospitals, research groups and individual radiology leaders.

## Gaps in radiological research and research education, current activities and future solutions

### Presence of radiology in basic research

Radiologists are often involved in the application and clinical assessment of technical innovation rather than performing their own laboratory-based research or epidemiological studies. Innovation-related research is important and should be continued. However, in order to have an impact on funding agencies and major university programmes, radiologists must be involved in basic research with an emphasis on both biological and physical topics.

Education relating to basic research is primarily the responsibility of universities. There are a number of M.D./Ph.D. programmes related to radiological topics throughout Europe [3], but also shorter programmes such as summer schools (http://www.excite.ethz.ch/). Radiological societies probably cannot (and should not) influence university programmes directly. However, radiology chairpersons should actively and systematically be involved in research activities by the scientific societies. There may be a need for an association of university radiologists or a research-oriented academy in order to increase participation of university hospitals in research education. Such an entity may best work under the umbrella of the ESR to avoid duplication of effort. The US-based Association of University Radiologists (AUR) and its affiliated groups such as the Radiology Research Alliance (RRA) (http://aur.org/AffinityGroups/http://aur.org/AffinityGroups/) may represent an interesting example.

The ESR is already very active in basic research, mainly through its EIBIR (European Institute for Biomedical Imaging Research, www.eibir.org) organisation. The ESR could further contribute to basic and clinical research activities through further activities driven by its research committee in the fields of imaging biomarkers, standardisation, imaging biobanks and personalised medicine. Studies resulting from this strategic planning should be operated by EIBIR. On the practical side, the European Congress of Radiology (ECR), European School of Radiology (ESOR) and the European subspecialty societies may contribute by systematically including basic research in their programmes and activities, adapted to the educational levels of their main audiences. The authors are aware that sessions and courses including basic research are not mainstream and would have to be tailored carefully to the needs of the general radiology audience in order to fulfil its goals; for example, by providing interactive courses including both basic researchers and clinical radiologists, similar to courses provided by other societies, including the International Society for Magnetic Resonance in Medicine (ISMRM).

The training in radiology through a medical residency system has proved to provide an adequate formation to the future specialists in clinical radiology. However, residency programmes tend to be tilted towards an almost purely clinical approach, which can lead to a deficit in research education.

It is mandatory to include an adequate training in the basic principles of research and evidence-based medicine in a thorough and comprehensive radiology curriculum in order to train a complete radiologist. This research education includes a satisfactory formation in the elements of the scientific methods, understanding the basic statistical knowledge in order to critically read and assess scientific publications, and an introduction to the design of research studies and scientific publishing (scientific posters and communications, articles). Training should be carried out under the supervision of the pertinent experienced trainers, and with an adequate degree of supervision appropriate to the status and training year of the trainee [4–6]. In that sense, the European Training Curriculum for Radiology developed by the ESR provides a valuable template to assure a basic education.

The radiology residency’s final goal is to develop a complete specialist in general radiology and all trainees should be completely trained in all fields and disciplines in order to achieve this target. However, radiology training can also be a starting point to those trainees who would rather focus on a research-oriented career than on a more clinically-oriented practice. Mentors and tutors of the radiology departments in charge of radiology trainees can help in orienting and guiding those trainees without losing the principle of an adequate and general formation.

Research is a discipline that benefits from scientific exchange and, furthermore, taking into consideration the increasingly global society we live in, where international interchange is a usual practice. As a global and international society, the ESR and its associated institutions could take a leading role in providing a frame for scientific and research exchange programmes for radiology trainees.

### Radiology-specific research versus research services

Radiology is important for the research undertaken by other disciplines; for example, as a service by acquiring, evaluating, communicating and storing imaging biomarker data. Such activities contribute to research in radiology as they connect radiologists to a research network and transfer knowledge from statistical methods, quality control, good manufacturing practice (GMP), industry-based research and university-based research into radiology. However, it does not directly give the radiologists a substantial role in basic research. Research education needs to take this important source of knowledge into account and needs to provide basic knowledge in the relevant topics.

Equally important, radiological research activities should not remain limited to a service to clinical partners, but original questions should arise from radiology itself, and the respective research methodology must be taught to radiologists. Radiology needs to broaden its research activities, from animal studies to randomised clinical trials and thus obtain an autonomous role in basic and clinical research. Radiology is also well suited to epidemiological research; for example, through the management of image databases and by providing and developing biomarkers. In order to survive in the current and future healthcare environment, radiology may not be sufficiently active in providing its own data on patient outcomes related to radiological diagnoses and therapeutic interventions. Various training opportunities by the ESR and EIBIR represent the basic framework for future development.

### New research topics

New research topics develop continuously, and radiology as well as radiological education must follow such developments. New radiological research topics may originate from clinical questions. Examples include new surgical techniques requiring preoperative imaging for proper indication, surgical planning, early detection of complications and follow-up. Similar questions arise in non-surgical disciplines; for instance, after the introduction of new medication. There are also more general trends requiring imaging support, such as the increased interest in obesity, lifestyle issues and ageing. Imaging biomarkers are important in many disciplines, such as oncology, cardiology, rheumatology, liver surgery and orthopaedic surgery. Personalised medicine is another area of potential growth where radiology will have an important role.

There may also be research opportunities at the intersection with other disciplines, sometimes unexpectedly. Behavioural research may involve radiology; for example, in the assessment of the role of training, of fatigue or of incentive drivers. Imaging of new materials provided by material science is another example of where radiologists are commonly involved; for instance, in the assessment of new orthopaedic or cardiac implants.

Radiology needs to monitor such new opportunities and draw its own conclusions in a systematic fashion. First of all, radiologists, particularly those associated with academic institutions, need knowledge of current basic research as well as clinical research and what topics are becoming active. We should not rely simply on the interest of a few individuals but we need professional background as well as organisational representation. Professional background can be obtained by dual education, such as M.D./Ph.D. programs, or dual appointments, such as physicists, biologists or clinicians employed in radiology departments. Radiological societies, above all the ESR, should firmly anchor basic research topics in their programme. Individual radiology leaders should lobby their universities to provide educational and research programs, such as M.D./Ph.D. programs, basic research summer schools and joint series of lectures. Those associated with journals should try to attract and support publication of basic research papers. Although such work may be of less interest to many readers, it may foster important discussions. All academically active radiologists can contribute to this process, from serving as a reviewer for journals up to the editors. Societies also have the power to promote basic research through prizes and through the construction of their meeting programs.

### Attitude

The commonly cited “Generation Y”, those born between 1980 and 2000, may have a reputation which is worse than it actually deserves. However, limited working hours, predictable careers and meaningful work contents may not favour research activities alongside a busy clinical schedule [7]. At the same time, some senior radiologists may not see research and research education as important as the ESR Working Group on Research Educations might hope. When time and other resources are limited, research competes with clinical work and clinical training and may easily lose.

In turn, the many positive facets of Generation Y should be addressed. Empathy and true interest in meaningful work are typical for this generation and may lead to an extra effort in research. To convince Generation Y of the importance of research and research education for the future of their discipline is crucial. Most important in this process are radiology leaders and other role models, such as basic researchers, who should promote the concept of radiology as a research-oriented discipline.

The even younger “Generation Z” (digital natives, born after 2000) will soon start to enter medical school and will expect systematic use of digital technologies for teaching and research. Similar to Generation Y, Generation Z is eager to learn and to achieve a meaningful goal. This general motivation should be reinforced by radiology training programs that need to focus on the science aspects of the specialty.

Meaningful work, application of computer sciences, and adaptation of time schedules are in the hands of radiology leaders, universities and research societies. The ESR should emphasise the importance of research and research education, whilst recognising academic leaders within society.

### Career planning

The previously mentioned questionnaires indicate that career planning is an important topic and that relevant gaps are perceived. Training currently does not sufficiently emphasise grant applications, research ethics or required basic science. To promote and teach such activities may be too demanding for some teaching institutions, especially in a non-academic setting, but even for some university institutions. Family-friendly working models may not be sufficiently available in many countries and institutions, further impinging on motivation to perform research.

In Europe, residency programs and other curricula tend to be less formalised than, for example, in the USA. Within the research education working group there was no consensus regarding the effect of such lack of formalisation: does this lead to a waste of time and loss of focus, or is this academic freedom or avoidance of unnecessary bureaucracy? Generation Y may give the answer, as they like predictable career paths and select residency programs according to factors such as reliability and predictability.

Admittedly, the lower European formalisation is not proven to be inferior to the USA system; for instance, when looking at research productivity [8]. However, formal systems are better able to detect and correct underperforming individuals and institutions.

The risky business of research projects can be made more predictable by several measures; training in methodology, providing statistical and other scientific counselling, sharing the risk by working in research groups with more than one project, regular meetings and reporting, in order to identify deviation from the planned track. Day-to-day management of research careers will help young researchers in their longer education to attain promotion to staff positions. One possibility is to offer clinical fast track for those committed to research with strictly organised access to topics with limited training positions; for example, interventional radiology training. This is the task of radiology chairs but may be facilitated by carefully adapting the European and national training charters in order to better accommodate such career tracks.

Another important topic is the “pipeline leakage”, with capable researchers leaving the scientific track before reaching senior staff positions. Systematic career planning by radiology chairs is probably the best way to avoid preventable loss of careers. This includes scouting for open positions for the younger, not yet connected radiologists, as well as preparation for applications to leading positions.

Such promotion of research is not an easy task. Clinical routine work remains the basis of all radiology. To find a way to promote researchers without frustrating clinical radiologists remains an art of leadership.

### Incentives

There must be intrinsic motivation to perform research and to participate in research education in order to enjoy working as a researcher. The willingness to contribute to human knowledge, to develop the discipline of radiology and to foster patient wellbeing are strong intrinsic motivations [9]. To identify such individuals is another important task for radiology leaders, possibly supported by the hospitals’ human resources departments. However, even highly motivated residents need some incentive not to lag behind purely clinical colleagues of the same age. Clinicians may be promoted to clinical staff physician earlier than researchers with their longer education path. In the long run, trainees may also look at salaries in the academic versus private practice system. The impact of lower salaries in the academic system must be offset not only by intrinsic motivation but also by extrinsic factors such as academic rewards. The responsibilities are with the chairpersons of the departments of radiology who should ensure that salaries are similar to clinical salaries in their own institutions and not too low in comparison to other institutions competing for the same radiologists. They cannot do this without the help of programmes for financing of protected time or support personnel. In addition, universities (and faculties of medicine) as well as scientific societies have means to compensate active research by academic promotion and by invitations to speak at prestigious meetings. When chairpersons make decisions regarding congress participation, research activities should be a top criterion.

### Gender issues

The proportion of women in medical school, in residency and more recently in staff positions is increasing. Childbearing at least for a certain period of time does not allow to work full time, may lead to reduced research in favour of clinical activity and may reduce the status of women who wish to remain in the competitive research environment. Women may be less inclined than men to choose a research career, and the brain drain from the academic system is more pronounced for women than men (http://www.gleichstellung.uzh.ch/politik/gleichstellungsmonitoring/GLM_Datentabellen_2013.pdf). At the University of Zurich, 1991–1993, 46.8 % of newly registered students were women. However, in 2013, the percentage of female full professors was 19.5 % instead of the expected half of all positions at University of Zurich. Another reason to formally address women in radiology relates to the fact that a mixed leadership has been shown to be more successful than single-gender groups. When reviewed from an economical point of view, net income growth for companies with women board members has averaged 14 % over the past 6 years compared with 10 % for those with no female board representation [10].

To keep the increasingly female radiology workforce in academic institutions is a standard task for radiology chairs, hospitals and universities. Quota may not be the best way to reach this but may be taken into consideration if voluntary programmes are not successful. However, the efforts of each individual staff member are as important. They provide role models, may actively recruit female candidates for research career tracks, and ensure that females are adequately represented in search committees. A discussion of this topic is provided in the bestselling book “Lean in” by Facebook’s COO, Sheryl Sandberg [11], and in the multi-editor book, “The Glass Ceiling in the 21st Century: Understand Barriers to Gender Equality” [12].

### Financing of research education

Funding constraints have occasionally been noted in the ESR research committee questionnaires. Indeed, training in general and research education specifically must compete with tighter budgets and stricter resource allocation models such as diagnosis-related groups, leaving no or little room for cross-subsidising research and teaching by healthcare funds.

Even under economic pressure, chairpersons have means to promote research. Workload is not always evenly distributed during the day, during the week or during all seasons. There tend to be low activity periods in clinical radiology; for instance, in the mornings before patients seen in outpatient clinics appear in the radiology department, or in summer when elective hospitalisations may be slower. Also, there are university programmes and private foundations willing to finance protected time. There is also a leadership aspect: such resources may be attributed in a competitive fashion. Radiology leaders are responsible for insisting on such efforts especially during periods of frustrations after rejected applications. Universities, societies such as the ESR, the European Union and industry may assist in efforts to finance research and research education. They may provide grants for protected time, research fellowships, summer schools, courses and other activities promoting research and research education.

### Variability

The position and the organisation of research education varies between countries but also between institutions within the same country. For students and level 1 residents (years 1–3 of the European curriculum) (www.myesr.org/html/img/pool/ESR_2014_ESR-EuropeanTrainingCurriculum_web_Dec.pdf), there is minimal compulsory research education specific to radiology. However, voluntary courses are often available even at these early levels. Many countries and individual universities provide M.D./Ph.D. programmes as well as shorter courses for topics such as good clinical practice courses (GCP). Training on the job and participation at society meetings such as the ECR are also often used as a means to promote research education. Variability between institutions is not necessarily a bad thing but also the expression of the freedom of research. However, it means that some of the potential to recruit future researchers is not fully realised. Nevertheless, research education is too important not to use the potential of all capable individuals. Institutions with less active programmes and countries with a less formalised research programmes and research education should agree to minimal activities. European societies play an important role through strategic statements, rules and regulations such as the European Training Charter, as well as their educational activities. Teach the teachers programmes may assist in reducing variability between programmes and countries, and are probably underused in radiology.

## Conclusions

Research education is the pacemaker for research-driven radiology of the future. This paper investigates how current gaps can be filled in research education and training. There are no easy solutions, but to advance is well within the remit and capacity of the various players in the field, from the ESR and its committees to the individual radiologist. Many means are already available but may need to be more systematically applied, existing structures such as EIBIR need to be strengthened, and leaders need to consistently and unequivocally emphasise the role of research for radiology.

## References

[1] Sardanelli F, Hunink MG, Gilbert FJ, Di Leo G, Krestin GP (2010). Evidence-based radiology: why and how?. Eur Radiol.

[2] European Society of Radiology (ESR) (2012). Education in research: results of a survey commissioned by the research committee of the European Society of Radiology. Insights Imaging.

[3] European Society of Radiology (ESR) (2013). MD PhD programmes with relevance to imaging. Results from a European survey. Insights Imaging.

[4] Barker CF (2013). Making imaging research a part of radiology resident training. Acad Radiol.

[5] Costello JR, Mullins ME, Votaw JR, Karolyi DR, Kalb B, Gonzales P (2013). Establishing a new radiology residency research track. Acad Radiol.

[6] Sundgren PC (2012). Mentoring radiology residents in clinical and translational research. Acad Radiol.

[7] The Economist (2013) Generations in the workplace. Winning the generation game. The Economist Newspaper Limited, London

[8] Spitzmueller D, Hodler J, Seifert B, Zanetti M (2010). Radiological research activity 1998–2007: relationship to gross domestic product, health expenditure and public expenditure on education. Insights Imaging.

[9] Buddeberg-Fischer B, Christen S, Weishaupt D, Hoffmann A, Kubik-Huch RA (2011). Professional satisfaction of radiologists in Switzerland. Swiss Med Wkly.

[10] Credit Suisse Research Institute, Mary Curtis Christine Schmid, Marion Struber. Editorial deadline, 13 July 2012, https://www.credit-suisse.com/newsletter/doc/gender_diversity.pdf

[11] Sandberg S (2013) Lean in: women, work, and the will to lead. Alfred A. Knopf, New York

[12] Barreto M, Ryan MK, Schmitt MT (2009) The glass ceiling in the 21st century: understanding barriers to gender equality. American Psychological Association, Washington

